# Noise logic with an InGaN/SiN_*x*_/Si uniband diode photodetector

**DOI:** 10.1038/s41598-022-12481-1

**Published:** 2022-05-19

**Authors:** Jiaxun Song, Richard Nötzel

**Affiliations:** 1 Guangdong Provincial Key Laboratory of Optical Information Materials and Technology, South China Academy of Advanced Optoelectronics, South China Normal University, Guangzhou, 510006 People’s Republic of China; 2 National Center for International Research on Green Optoelectronics, South China Normal University, Guangzhou, 510006 People’s Republic of China

**Keywords:** Mathematics and computing, Applied physics

## Abstract

Noise logic is introduced by the wavelength dependent photocurrent noise of an InGaN/SiN_*x*_/Si uniband diode photodetector. A wavelength versus photocurrent noise discrimination map is constructed from the larger photocurrent noise for red light than that for green light. A minimum measurement time of four seconds is deduced from the standard deviation of the photocurrent noise for a safe wavelength distinction. A logic NOT gate is realized as representative with on or off red or green light as binary 1 or 0 inputs and the photocurrent noise above or below a defined threshold as binary 1 or 0 outputs.

At the heart of computation, and information processing in general is Boolean logic executed by logic gates^1–3^. Such logic gates have a wealth of physical realizations with various types of binary input signals and output defining the underlying type of logic. At the forefront is electronic logic where the logic gates are electrical circuits containing transistors and diodes^4–8^. Photonic logic gates are built up of cavities and waveguides for all optical switching, which may contain active, non-linear materials such as atoms, molecules or quantum dots^9–15^. Switching in magnetic logic relies on magnetic hysteresis and can be performed in nanomagnet assemblies or magnetic multilayers with ferromagnetic or antiferromagnetic coupling^16–18^. In logic based on DNA and other bio- and macromolecules, configurational changes and molecular excitations, triggered by the addition of other substances such as nanoparticles or by other changes in the environment such as the exposure to light, or pH and temperature changes, can be utilized to perform the logic functions^19–31^. Neuron logic attempts to mimic the brain^32,33^. All these realizations of logic operations, however, suffer from noise, bringing about errors.

Here, we introduce noise logic using the wavelength dependent photocurrent noise of our InGaN/SiN_*x*_/Si uniband diode photodetector (PD)^34,35^, acting as the logic gate. The PD is operated in the self-powered mode at zero externally applied voltage. In Ref.^35^, all details of the fabrication and properties of the PD can be found, including wavelength dependent light/dark current–voltage measurements, photocurrent versus incident light power measurements, responsivity, detectivity, time response, photocurrent noise and discussion of the operating mechanism with band structure and photogenerated carrier transport.

Light of two different wavelengths is the input and the photocurrent noise is the output. To be more precise, the photocurrent noise is defined as the standard deviation of the photocurrent measured over time. The such defined photocurrent noise is used as the output signal to readout the logic operation of the logic gate in the proposed noise logic. Longer wavelength light, denoted as red light, is absorbed in the p-type Si substrate with bandgap energy of 1.1 eV while shorter wavelength, green light is absorbed in the n-type InGaN layer on top with 40% In content and 2 eV bandgap energy. Due to the unique band alignment of the conduction band of InGaN with In content around 40% with the valence band of Si, together with the insertion of the thin SiN_*x*_ interlayer, the noise of the ‘red photocurrent’ is markedly larger than the noise of the ‘green photocurrent’. This is because photogenerated minority electrons in Si tunnel through the SiN_*x*_ interlayer before energy relaxation while photogenerated minority holes in InGaN solely relax in energy and pass the SiN_*x*_ layer by diffusive transport.

We first establish a wavelength versus photocurrent noise discrimination map for red light with 808 nm wavelength and green light with 532 nm wavelength. These wavelengths best fall into the long- and short wavelengths regions to be distinguished. The wavelength dependent average photocurrent versus incident light power calibration curves then complete dual wavelength photodetection, proposed before^35^. A minimum measurement time of four seconds is deduced from the standard deviation of the photocurrent noise for a safe distinction of the wavelengths. With the red or green light on or off as binary 1 or 0 inputs, a logic NOT gate is demonstrated as representative with the total photocurrent noise above or below the defined threshold line as binary 1 or 0 outputs. Figure 1 shows again for clarity a scheme of the PD band structure, including the photogenerated carrier transport^35^, which involves diffusion, tunneling and energy relaxation, the measurement setup and the sample structure.

**Figure 1:**
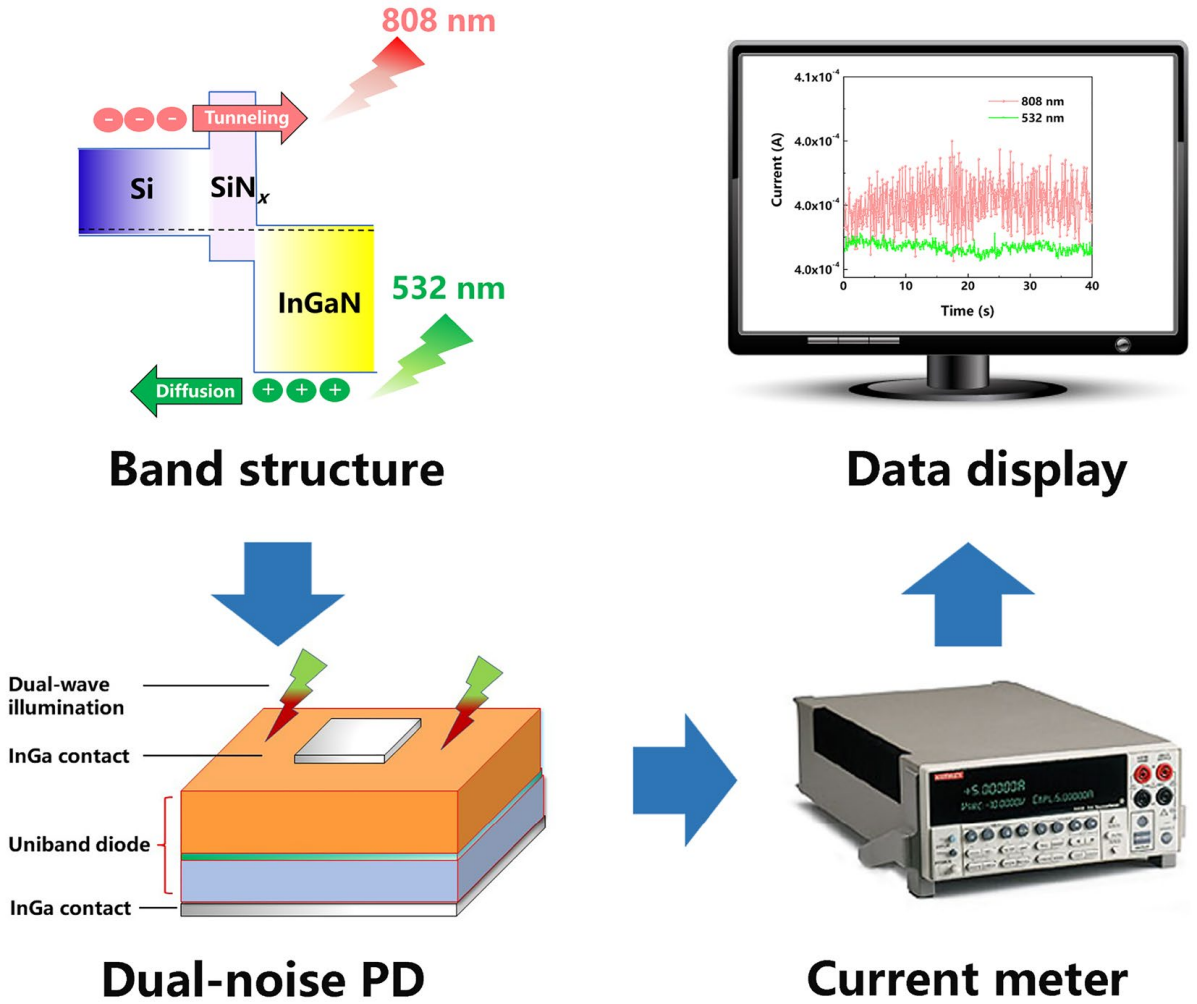
Experiment. Scheme of the PD band structure, measurement setup and sample structure.

To emphasize the significance of the noise logic, we generalize: any binary logic operation is based on a certain signal and a threshold to discriminate between two states of the signal. Noise is superimposed on any such signal and is detrimental. The paradigm change and advantage of noise logic, once a clear noise threshold can be defined, lies in the use of the always present noise as the output signal of a logic gate to perform logic operations.

## Results and discussion

**Figure 2:**
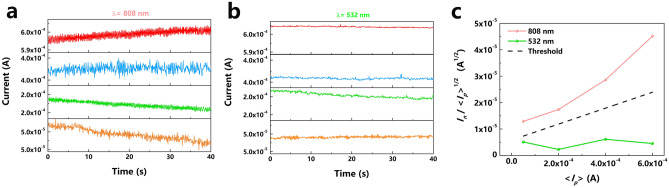
Dual-noise properties. (**a**,**b)** Photocurrent versus time measurements of the red and green photocurrents for four different average photocurrents. (**c)** Plots of the red and green noise currents divided by the respective square root of the average photocurrent as a function of the average photocurrent. The black dashed line indicates the threshold for the distinction of red and green light.

Figure 2a,b shows the photocurrent versus time measurements for the red (808 nm) and green (532 nm) incident light for four different average photocurrents. The measurement time is 40 s, including 500 sampling points for the sampling time of 80 ms. The four average red and green photocurrents are adjusted to the same values by the respective incident light power for a direct comparison of the red and green noise currents. Clearly, the red noise currents are consistently larger than the green noise currents, making possible the wavelength distinction. This is compiled in the wavelength versus noise current discrimination map for red light and green light shown in Fig. 2c. The red and green noise currents, divided by the square root of the red and green average photocurrents are plotted as a function of the average photocurrent. This plot is motivated by the shot noise current ${I}_{sn}=\sqrt{2e\langle {I}_{p}\rangle B}$, normalizing for the dependence on the average photocurrent, although the total noise current is dominated by the low-frequency *1/f* noise^35^. *I*_*sn*_ is the shot noise current, < *I*_*p*_ > is the average photocurrent, *B* is the detector bandwidth and *e* is the elementary charge. The black dashed line through the points half between the red and green noise currents defines the threshold for identifying the longer wavelengths in the region above—and the shorter wavelengths in the region below this threshold line.

**Figure 3:**
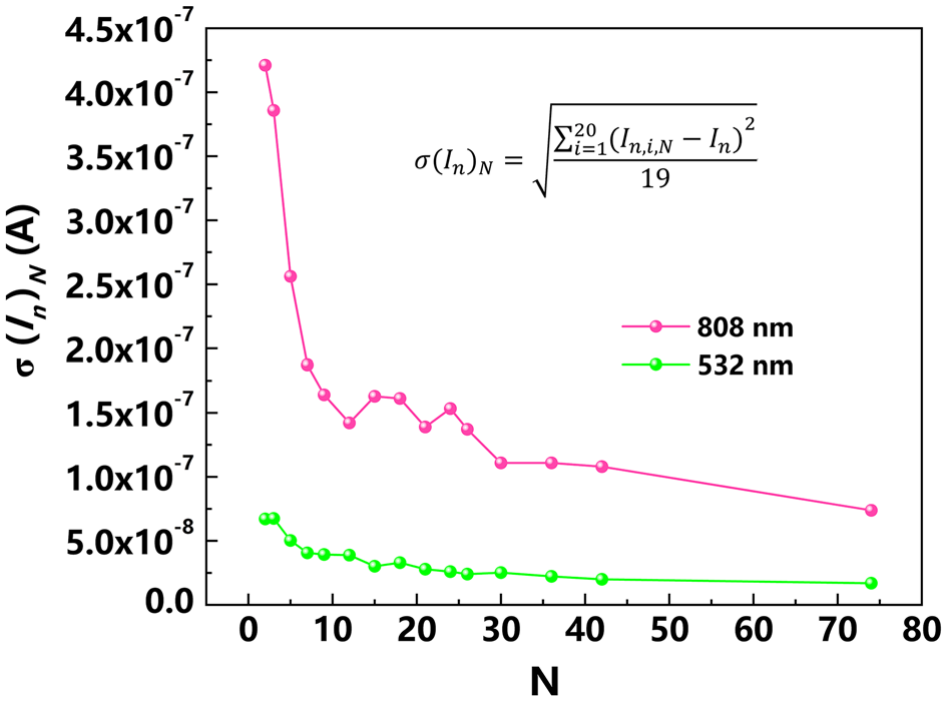
Noise statistics. Population standard deviation of the photocurrent noise for a fixed number of sampling points *N* as a function of *N*. The population standard deviation of the photocurrent noise for fixed *N, σ*(*I*_*n*_)_*N*_*,* is determined from the sample standard deviation of the photocurrent noise *I*_*n*,*i*,*N*_ for a sufficiently large number of 20 arbitrarily chosen windows *i* with the same *N*, according to the inserted formula. For the population mean photocurrent noise, the photocurrent noise *I*_*n*_ for the 40 s long measurement is taken.

Next, the minimum measurement time is deduced to safely distinguish between the red and green light, relying on the respective photocurrent noise. The photocurrent noise, defined as the standard deviation of the photocurrent measured over time, is a statistical property of the photocurrent, depending on the measured time interval. Therefore, using the photocurrent noise as signal, its accuracy and, hence, the ability to distinguish between the red- and green photocurrent noise signal increase with the measurement time or number of sampling points *N.* To be accurate, we need to determine as close as possible the population standard deviation of the photocurrent noise as a function of *N*, which itself is the sample standard deviation of the photocurrent for a certain *N*. From this, the minimum *N* or minimum measurement time is deduced such that the population standard deviation of the photocurrent noise is small enough to distinguish the red and green photocurrent noise signals. Underlying from statistics is, that the standard error of the sample mean is inversely proportional to the square root of the number of observations. The sample mean here is already the sample standard deviation of the original observable. The population standard deviation of the photocurrent noise *I*_*n*_ for a fixed *N* is written as *σ*(*I*_*n*_)_*N*_. And, again, decreases when *N* is increased. *σ*(*I*_*n*_)_*N*_ and its dependence on *N* are determined as follows: (i) Take 20 windows, each containing *N* sampling points. Distribute these 20 windows arbitrarily over the measured photocurrent curve. Here it is assumed that 20 windows are sufficient that the corresponding sample standard deviation of the photocurrent noise is sufficiently close to the population standard deviation. (ii) Calculate for each window the photocurrent noise *I*_*n*,*i*,*N*_, where *i* numbers the windows from 1 to 20. (iii) Calculate the sample standard deviation of *I*_*n*,*i*,*N*_ for the 20 windows taking as population mean the photocurrent noise *I*_*n*_ deduced from the 40 s long measurement with 500 sampling points. The result is *σ*(*I*_*n*_)_*N*_. (iv) Calculate *σ*(*I*_*n*_)_*N*_ for *N* from 1 to a sufficiently large value and plot *σ*(*I*_*n*_)_*N*_ versus *N*. Such a plot of *σ*(*I*_*n*_)_*N*_ versus *N* is shown in Fig. 3 for the red and green noise currents for the average photocurrent of 0.4 mA. The formula for calculating the population standard deviation *σ*(*I*_*n*_)_*N*_, which is assumed to be close to the sample standard deviation of *I*_*n*,*i*,*N*_ is given in the inset. When converting N to the 80 ms sampling time, Fig. 3 is a plot of the population standard deviation of the photocurrent noise as a function of the measurement time.

For a safe distinction of the light wavelengths with more than 99% probability, the difference of the red photocurrent noise divided by the square root of the average photocurrent and the dashed threshold line in Fig. 2c should be at least three times the standard deviation of the red photocurrent noise divided by the square root of the average photocurrent. This is the 3*σ* statistical confidence of 99.7% for which *N* must be larger than 50. For the 80 ms sampling time this gives a minimum measurement time of 4 s. It is straight forward to reduce the minimum measurement time by reducing the sampling time. Keeping the sampling time, the minimum measurement time can be reduced at cost of the statistical confidence. For 2*σ* statistical confidence of 95%, which is certainly acceptable, *N* must be larger than 20 and the minimum measurement time is 1.6 s. For 1*σ* statistical confidence of 68%, *N* must be larger than 8 and the minimum measurement time is 0.6 s.

**Figure 4:**
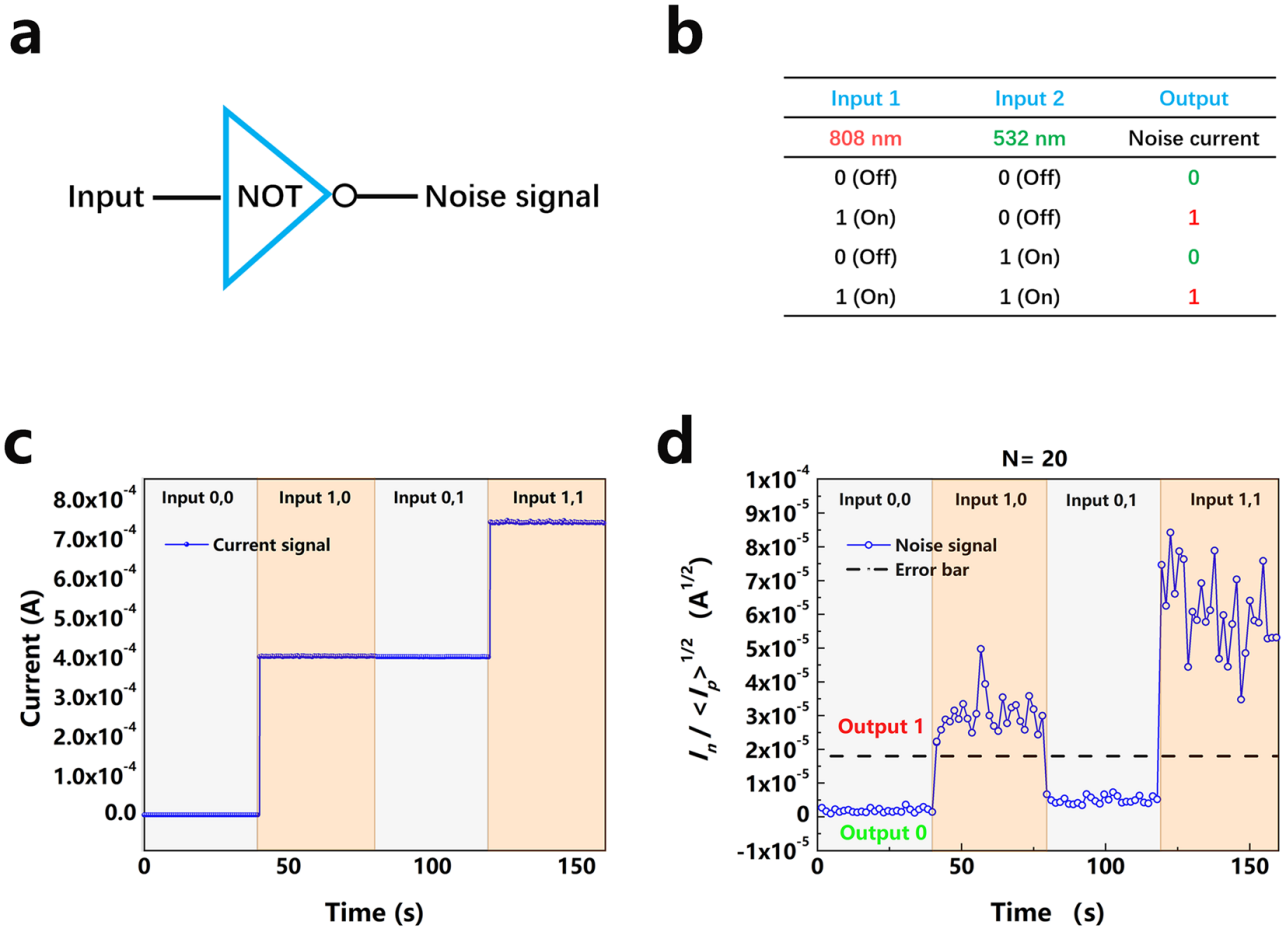
Noise logic demonstration. (**a**) Scheme of the logic NOT gate. (**b**) Truth table. (**c**) Average photocurrent for a sequence of logic inputs, as indicated. The red and green average photocurrents are 0.4 mA. (**d**) Logic photocurrent noise output 1 above and 0 below the black dashed threshold line for the same sequence of logic inputs.

As a representative of the derived noise logic, a logic NOT gate is demonstrated. The two inputs, input 1 and input 2, are defined by the red and green light, respectively. The on state represents the logic 1 and the off state represents the logic 0. As logic 1 or 0 outputs, the total photocurrent noise is taken. The logic 1 output is above the threshold line given in Fig. 2c and the logic 0 output is below the threshold line. The logic NOT gate is schematically drawn in Fig. 4a with the corresponding truth table in Fig. 4b.

Figure 4c depicts the photocurrent as a function of time for a sequence of logic (0,0), (1,0), (0,1) and (1,1) inputs, as indicated. The average photocurrent for both the red and green light is adjusted to 0.4 mA. Figure 4d shows the logic photocurrent noise output for the same sequence of logic inputs. The photocurrent noise divided by the square root of the average photocurrent above the black dashed threshold line in Fig. 2c defines the logic 1 and below the black dashed threshold line the logic 0. The total measurement time for each input is 40 s and each point results from an average over 1.6 s, i.e., *N* = 20 sampling points, choosing for the 2σ statistical confidence of 95%. This guarantees a sufficiently small standard deviation of the noise current and, hence, a definite distinction of the different logic outputs for nearly error free logic operations.

## Conclusions

In summary, we have demonstrated noise logic by exploiting the wavelength dependent photocurrent noise of an InGaN/SiN_*x*_/Si uniband diode PD. From the distinctly larger photocurrent noise for longer wavelength red light than for shorter wavelength green light, a wavelength versus noise current discrimination map was constructed. A minimum measurement time of four seconds was deduced from the standard deviation of the photocurrent noise for a safe wavelength distinction. A logic NOT gate was realized with the red or green light on or off as binary 1 or 0 inputs and the photocurrent noise above or below threshold as binary 1 or 0 outputs.

## Methods

### Growth

The In_0.4_Ga_0.6_ N layers were grown by plasma-assisted molecular beam epitaxy (PA-MBE) on p-type Si (111) substrates. Prior to growth, the Si substrates were etched in 10% HF solution for 1 min to remove the native oxide from the surface. The cleaned substrates were transferred into the load chamber and degassed for 30 min at 300 °C. Then, the substrates were transferred into the growth chamber and annealed at 900 °C (thermocouple reading) to remove residual native oxide. The thin SiN_*x*_ layer was formed at the same temperature by exposing the Si substrate surface to active N flux with radio-frequency (RF) power of the RF N plasma source of 350 W and 1.7 standard cubic centimetres per minute (sccm) molecular N_2_ flow for 20 min. For the growth of InGaN, the substrate temperature was reduced to 620 °C and the N plasma source settings were 220 W and 1.2 sccm. The In and Ga effusion cell temperatures were 814 and 845 °C with the In and Ga beam equivalent pressures of 1.05 × 10^−7^ and 7.47 × 10^−8^ Torr, respectively. The growth time was 1 h without growth interruptions.

### PD fabrication and measurements

The InGaN/SiN_*x*_/Si uniband diode PD was fabricated by deposition of Ga-In eutectic droplets on the InGaN layer and the backside of the Si substrate. The photocurrent versus time measurements were performed with a Source Meter (Keithley-2400). The sampling time was 80 ms. Two diode lasers with wavelengths of 808 and 532 nm were chosen as light sources. All measurements were carried out at room temperature under ambient conditions.

## Data Availability

All data supporting the findings in the article are available from the corresponding authors on reasonable request.
